# Supplementary material for: Penifuranone A: A Novel Alkaloid from the Mangrove Endophytic Fungus Penicillium crustosum SCNU-F0006
Source: Int J Mol Sci. 2024 May 5;25(9):5032. doi: 10.3390/ijms25095032 (PMC11084256; doi:10.3390/ijms25095032)
Supplement: Supplementary file 1 [file ijms-25-05032-s001.zip › ijms-2966443-supplementary.pdf]

Supplementary Material

Penifuranone A: A Novel Alkaloid from the Mangrove Endophytic Fungus *Penicillium crustosum* SCNU-F0006

Hao Jia, Li Wu, Rongrong Liu, Jialin Li, Lingling Liu, Chen Chen, Junsen Li, Kai Zhang, Junjiang Liao and Yuhua Long *

Guangzhou Key Laboratory of Analytical Chemistry for Biomedicine, School of Chemistry, South China Normal University, Guangzhou 510006, China; haojia@m.scnu.edu.cn (H.J.); wuli@m.scnu.edu.cn (L.W.); rongrongliu@m.scnu.edu.cn (R.L.); jialinli@m.scnu.edu.cn (J.L.); linglingliu600@m.scnu.edu.cn (L.L.); chenchen2021@m.scnu.edu.cn (C.C.); junsenli@m.scnu.edu.cn (J.L.); zhangkai2021@m.scnu.edu.cn (K.Z.); junjiangliao0712@m.scnu.edu.cn (J.L.)

* Correspondence: longyh@scnu.edu.cn

Table of content

Figure S1. Compounds **1-4** and **1a** were analyzed by TLC at a wavelength of 254 nm. 3
Figure S2. Compounds **1-4** and **1a** are tested with Wagner's reagent($I_2 \cdot KI$) for coloration. 3
Figure S3. Parameter of TLC. 4
Figure S4. HPLC analysis of compound **1**. 5
Figure S5. UV spectrum of compound **1** (MeOH). 6
Figure S6. IR spectrum of compound **1** (KBr). 6
Figure S7. HRESIMS spectrum of compound **1**. 7
Figure S8. ^{1}H NMR (DMSO-d_6,600 MHz) spectrum of compound **1**. 7
Figure S9. ^{13}C NMR (DMSO-d_6,150 MHz) spectrum of compound **1**. 8
Figure S10. ^{1}H, ^{1}H- COSY (DMSO-d_6, 600 MHz) spectrum of compound **1**. 8
Figure S11. HSQC (DMSO-d_6,600 MHz) spectrum of compound **1**. 9
Figure S12. HMBC (DMSO-d_6,600 MHz) spectrum of compound **1**. 9
Figure S13. NOESY (DMSO-d_6,600 MHz) spectrum of compound **1**. 10
Figure S14. UV spectrum of compound **1a** (MeOH). 10
Figure S15. IR spectrum of compound **1a** (KBr). 11
Figure S16. HRESIMS spectrum of compound **1a**. 12
Figure S17. ^{1}H NMR ($CDCl_3$, 600 MHz) spectrum of compound **1a**. 12
Figure S18. ^{13}C NMR ($CDCl_3$,150 MHz) spectrum of compound **1a**. 13

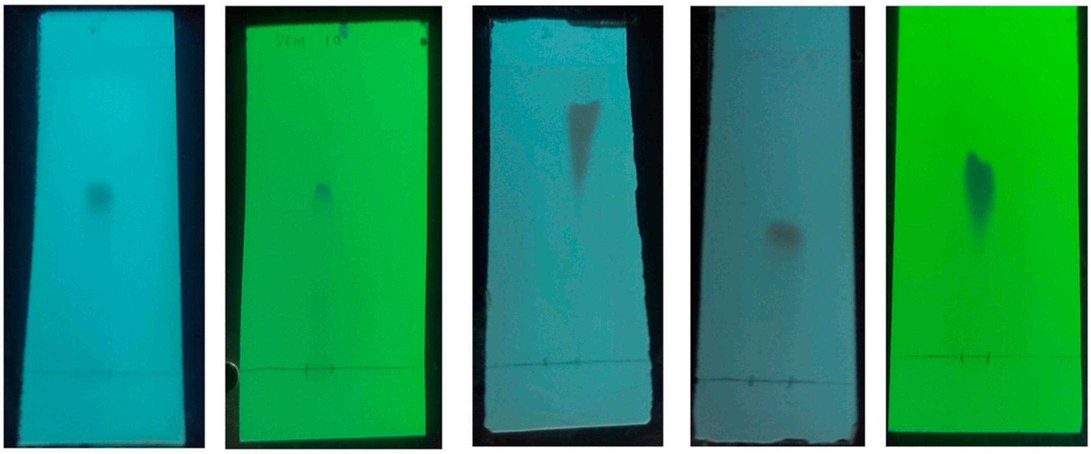

1 1a 2 3 4

Figure S1. Compounds **1-4** and **1a** were analyzed by TLC at a wavelength of 254 nm.

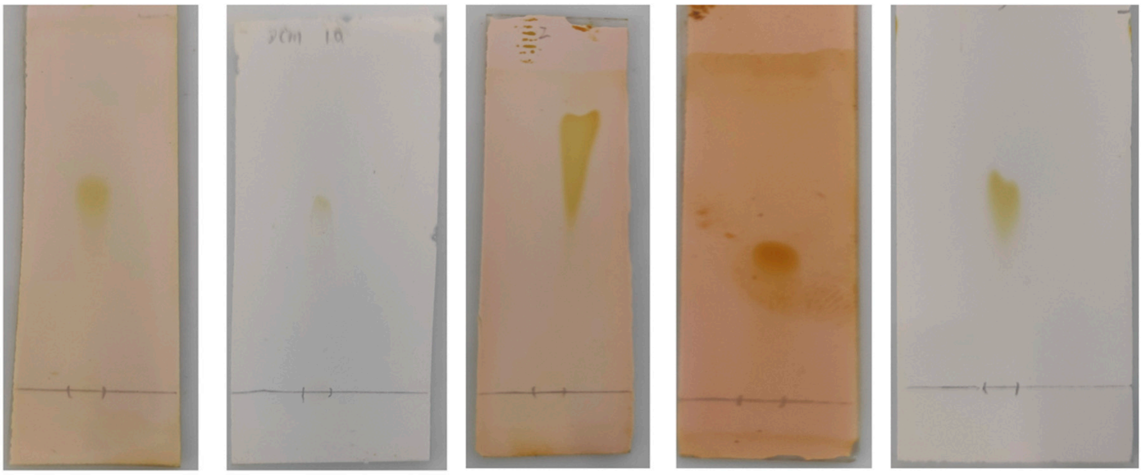

1 1a 2 3 4

Figure S2. Compounds **1-4** and **1a** are tested with Wagner's reagent(I_2 • KI) for coloration.

Compounds	Stationary Phase	Mobile Phase	Rf Value
1	TLC Silica gel 60 RP-18 F_{254}s	MeOH:H_2O=60:40	0.55
1a	TLC silica gel GF254 plates	Dichloromethane(DCM)	0.58
2	TLC Silica gel 60 RP-18 F_{254}s	MeOH:H_2O=70:30	0.77
3	TLC Silica gel 60 RP-18 F_{254}s	MeOH:H_2O=70:30	0.56
4	TLC silica gel GF254 plates	DCM:MeOH =60:6	0.52

Figure S3. Parameter of TLC.

SHIMADZU LabSolutions

Analysis Report

< Sample Information >

Sample : Compoud 1
Mobile phase : Methanol : Water = 60: 40
Injection volume : 20 uL
Date : 2024/4/20
Analyst: Hao Jia

< Chromatogram >

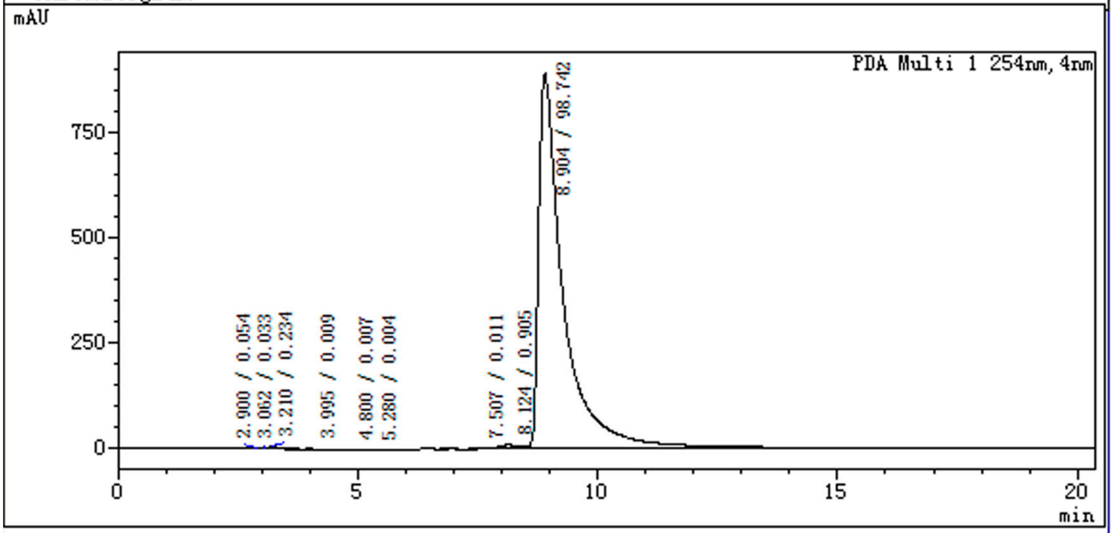

< Peak Table >

PDA Ch1 254nm					
Peak Number	Retention Time	Area	Area Percentage	Height	Height Percentage
1	2.900	17545	0.054	2125	0.233
2	3.062	10920	0.033	1951	0.214
3	3.210	76359	0.234	6425	0.704
4	3.995	2978	0.009	397	0.044
5	4.800	2227	0.007	188	0.021
6	5.280	1257	0.004	128	0.014
7	7.507	3594	0.011	308	0.034
8	8.124	295367	0.905	9159	1.004
9	8.904	32210811	98.742	891844	97.734
总计		32621058	100.000	912526	100.000

Figure S4. HPLC analysis of compound **1**.

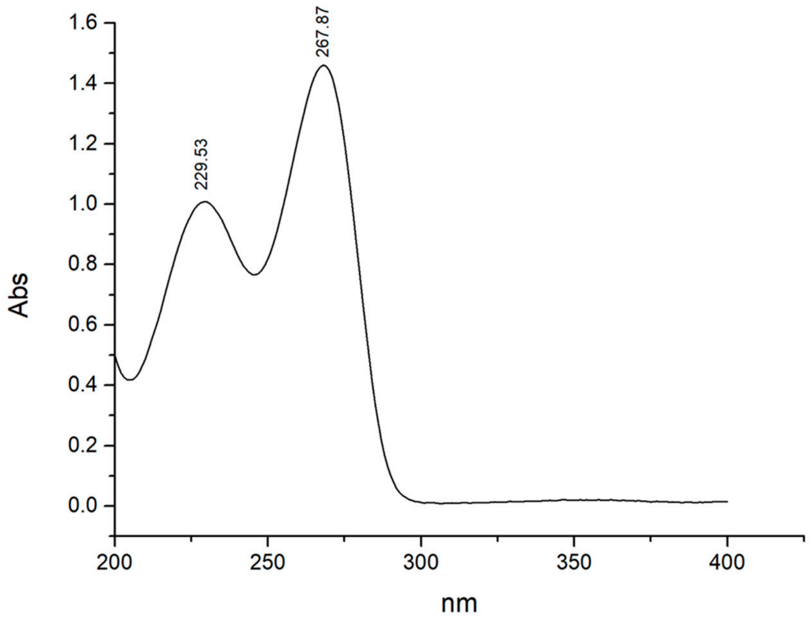

Figure S5. UV spectrum of compound **1** (MeOH).

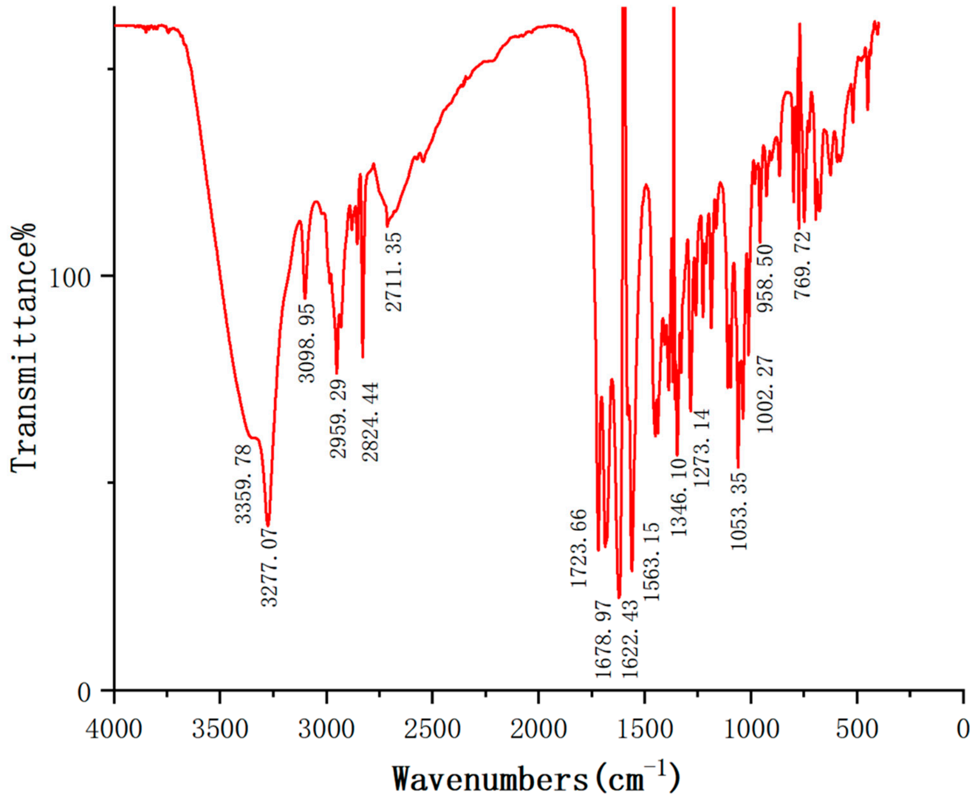

Figure S6. IR spectrum of compound **1** (KBr).

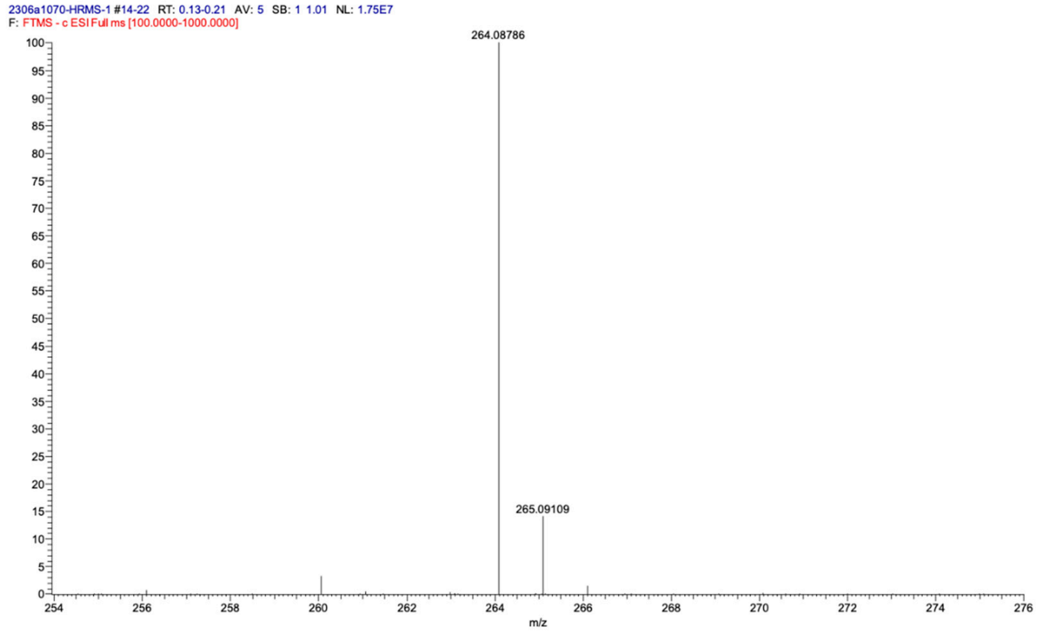

SPECTRUM - simulation :

m/z	Theo. Mass	Delta (ppm)	RDB equiv.	Composition
264.08786	264.08775	0.43	7.5	C13 H14 O5 N

Figure S7. HRESIMS spectrum of compound **1**.

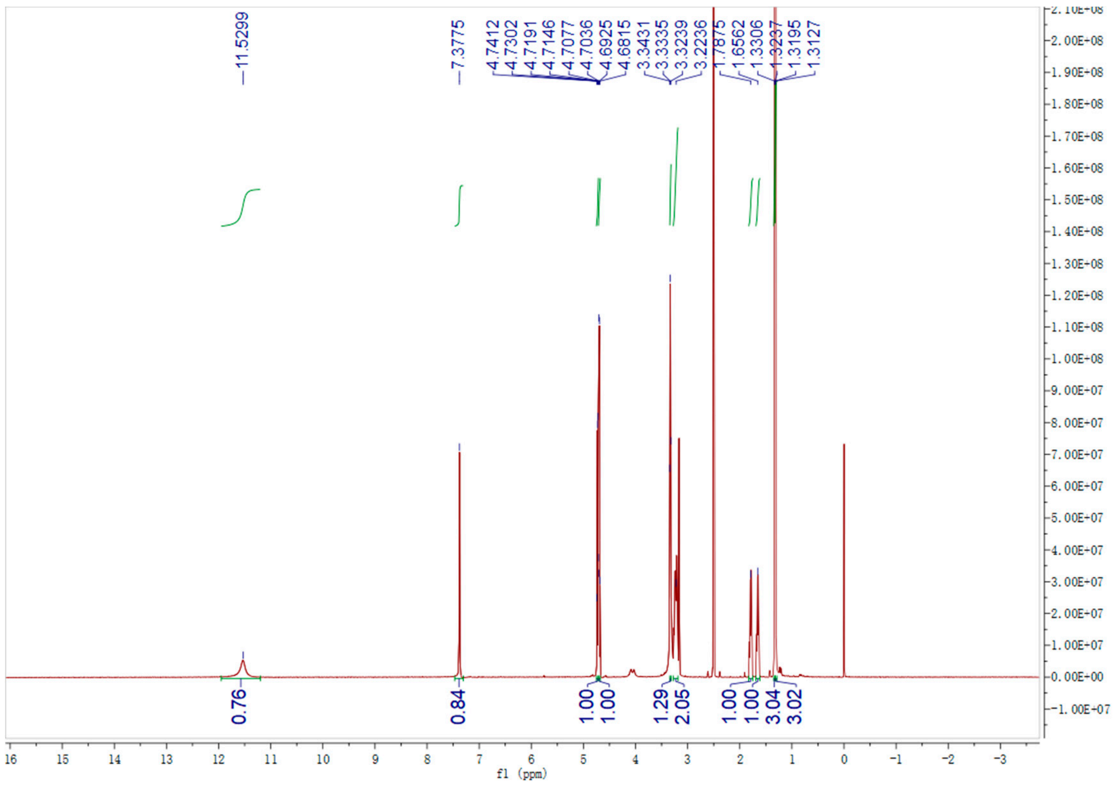

Figure S8. ^{1}H NMR (DMSO-d_6,600 MHz) spectrum of compound **1**.

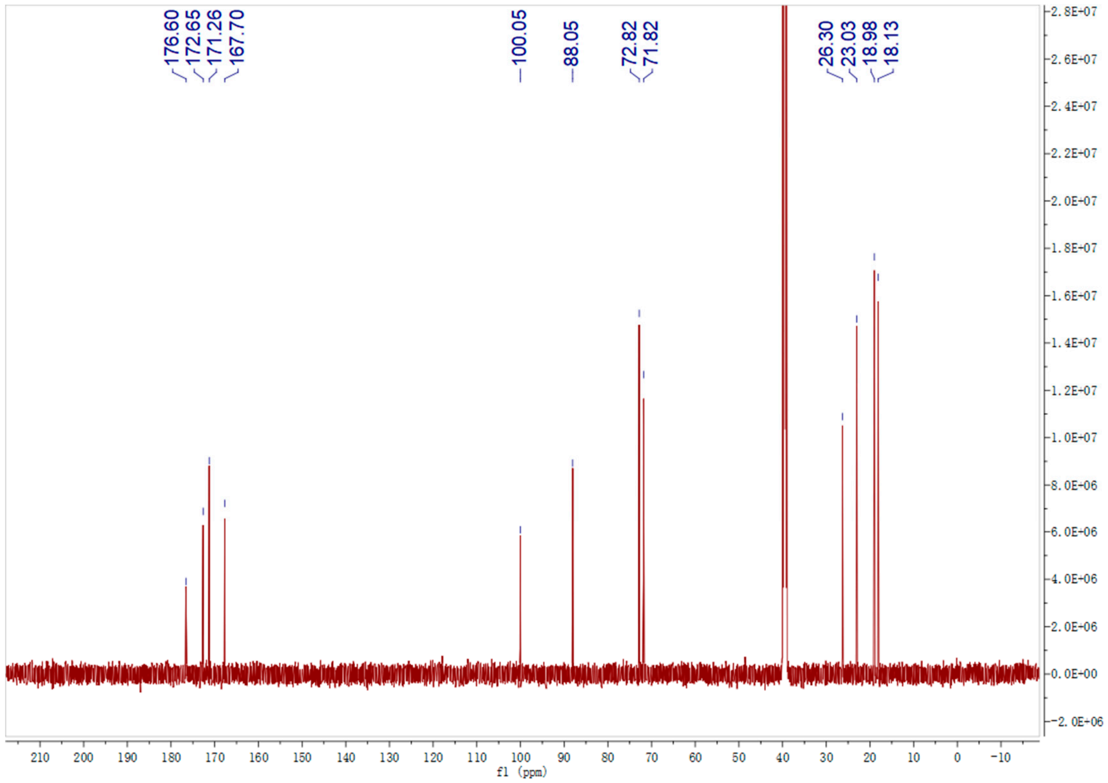

Figure S9. ^{13}C NMR (DMSO-d_6,150 MHz) spectrum of compound **1**.

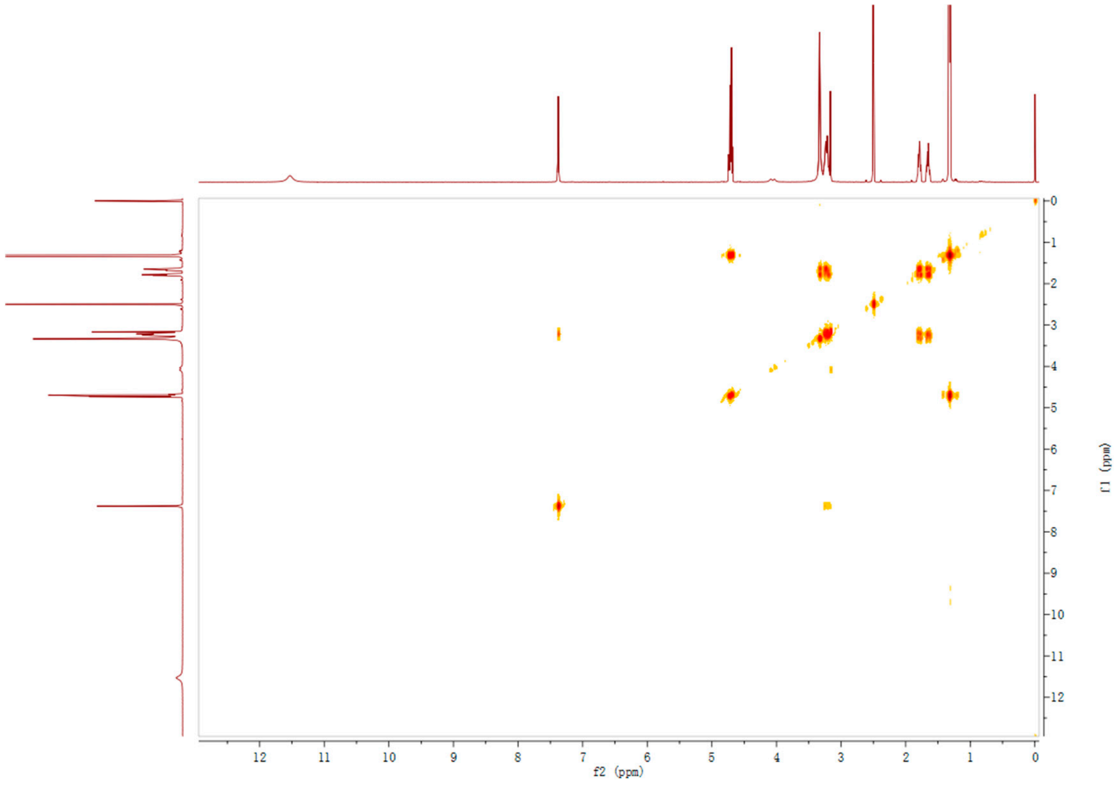

Figure S10. ^{1}H, ^{1}H- COSY (DMSO-d_6, 600 MHz) spectrum of compound **1**.

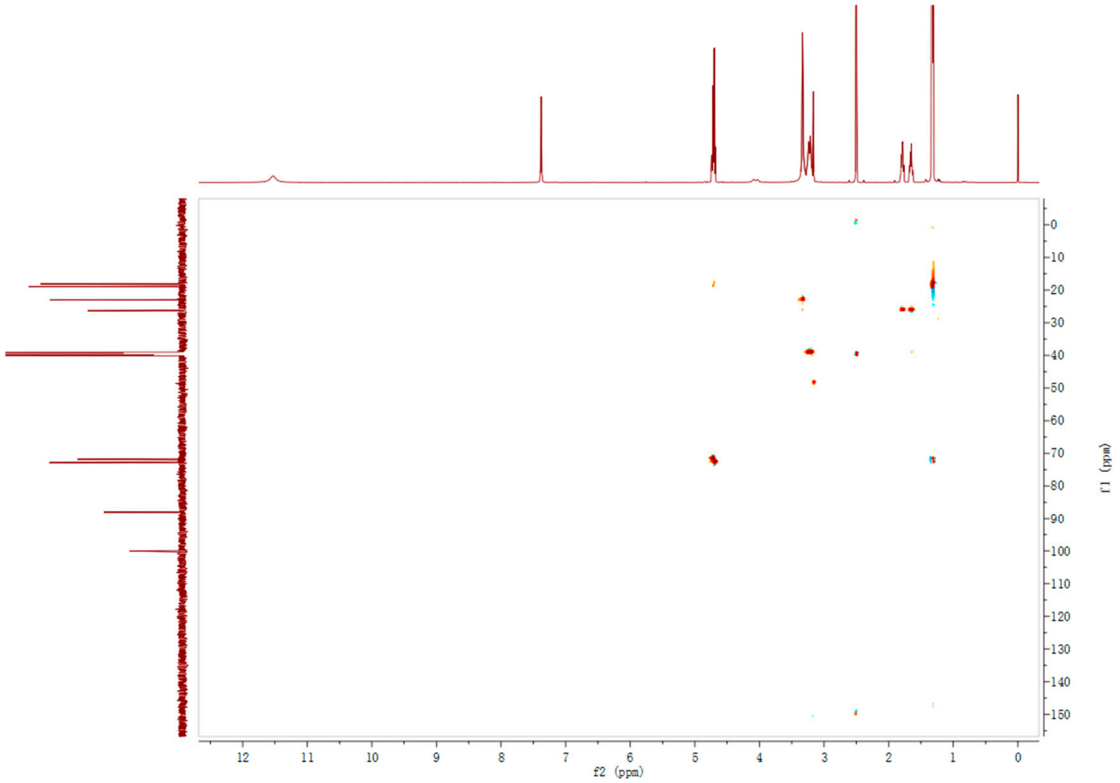

Figure S11. HSQC (DMSO-d_6,600 MHz) spectrum of compound **1**.

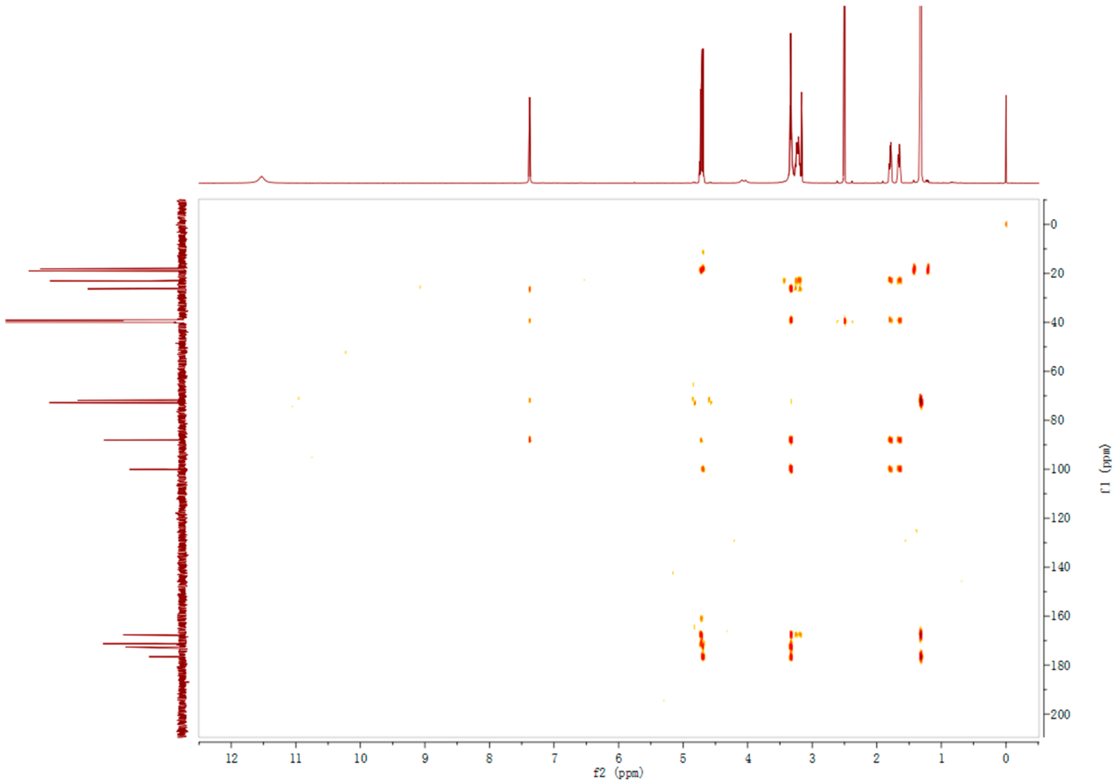

Figure S12. HMBC (DMSO-d_6,600 MHz) spectrum of compound **1**.

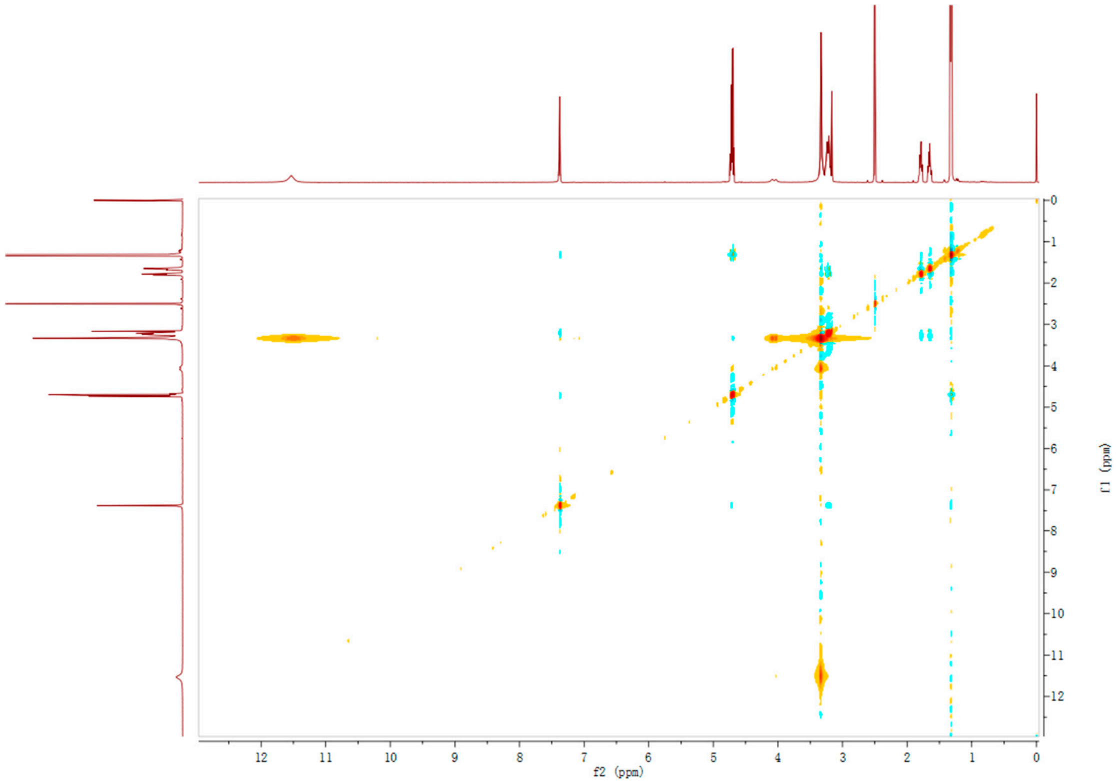

Figure S13. NOESY (DMSO-d_6,600 MHz) spectrum of compound **1**

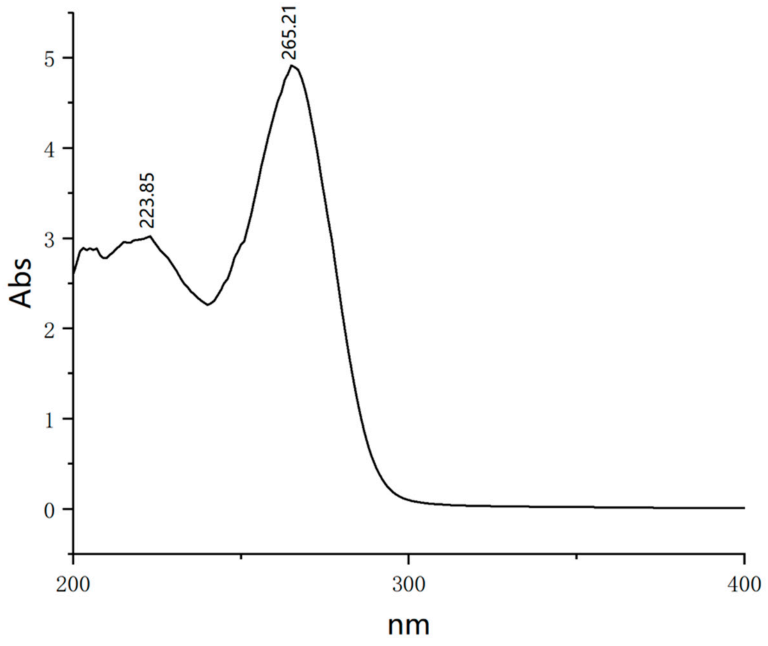

Figure S14. UV spectrum of compound **1a** (MeOH).

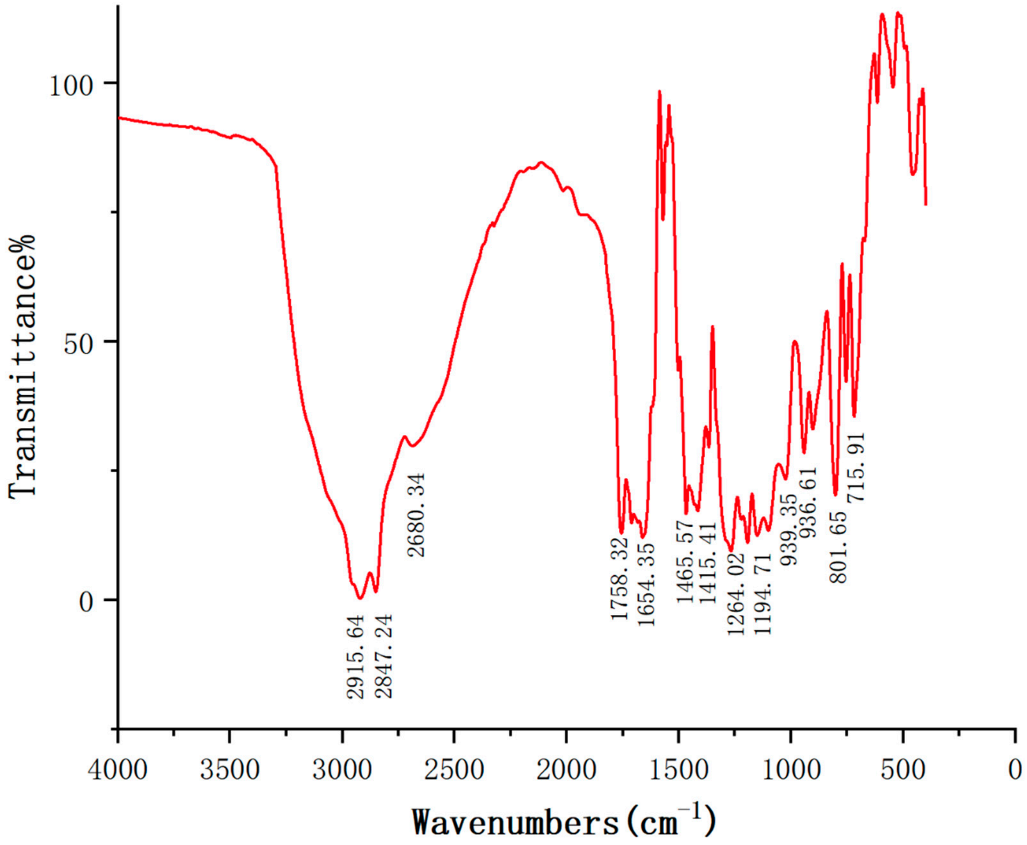

Figure S15. IR spectrum of compound **1a** (KBr).

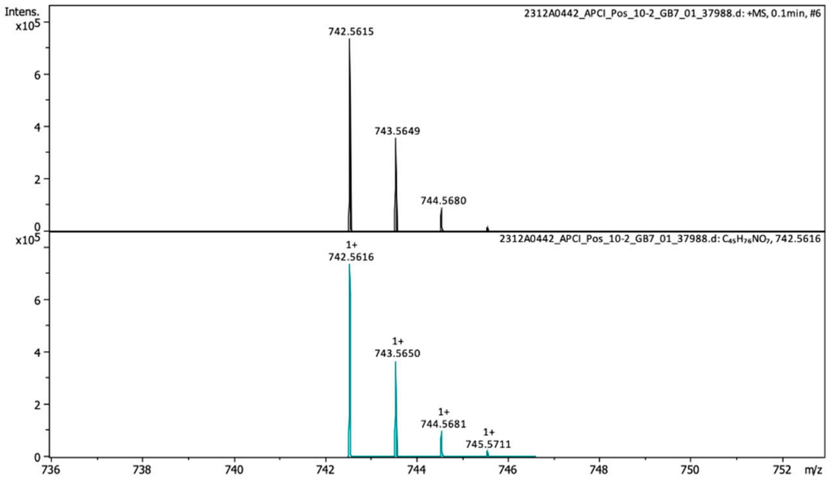

Meas. m/z	Ion Formula	m/z	z	err [ppm]	mSigma	rdb	e⁻ Conf	N-Rule
742.5615	C45H76NO7	742.5616	1+	0.1	12.1	9	even	ok

Figure S16. HRESIMS spectrum of compound **1a**.

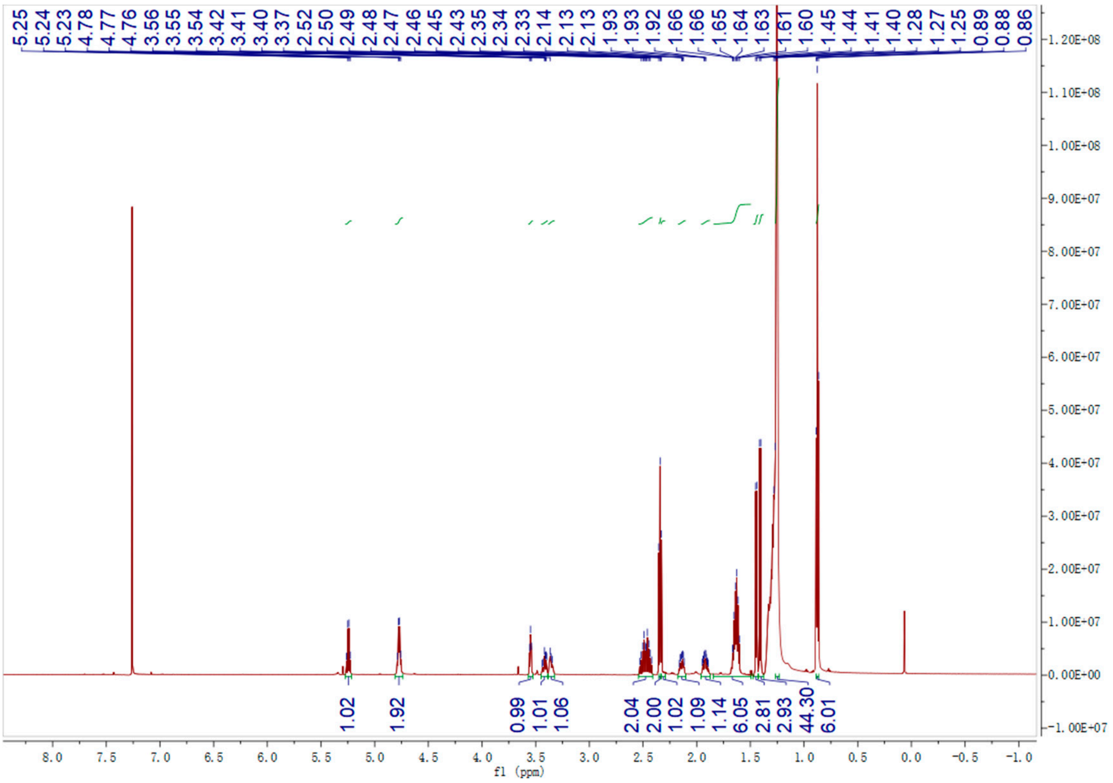

Figure S17. ^{1}H NMR ($CDCl_3$, 600 MHz) spectrum of compound **1a**.

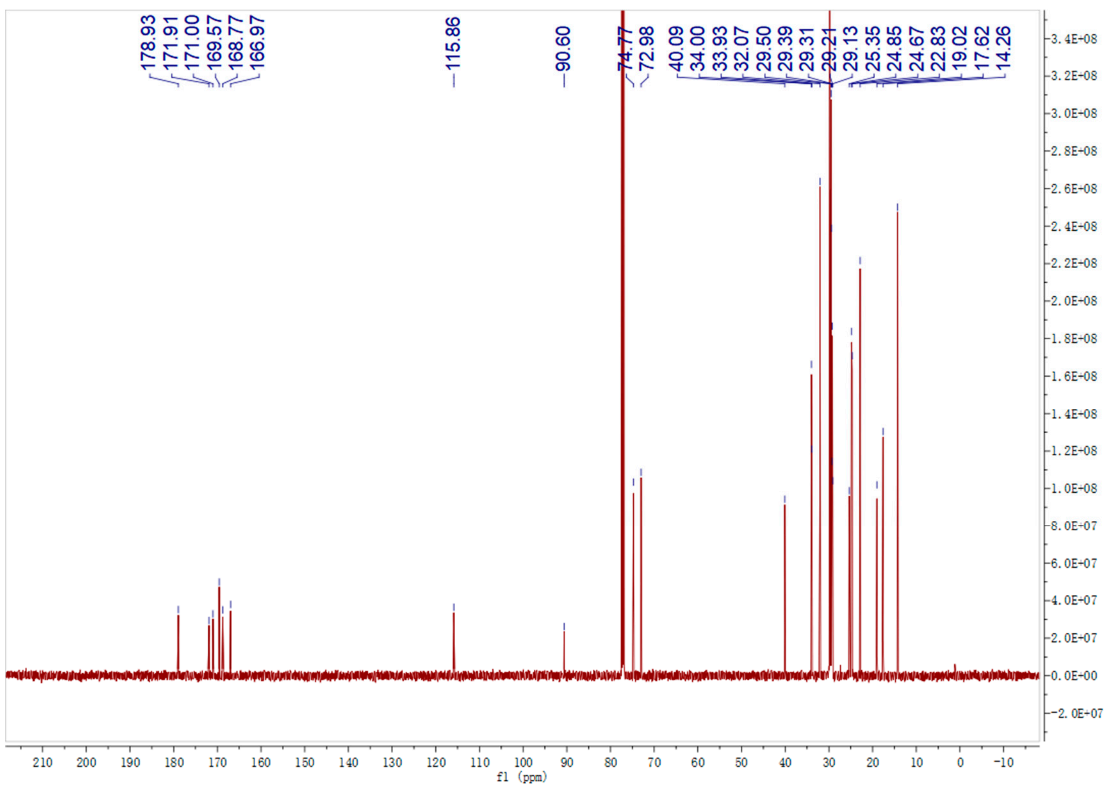

Figure S18. ^13^C NMR ($CDCl_3$,150 MHz) spectrum of compound **1a**.
